# Supplementary material for: Optically Driven Janus Microengine with Full Orbital Motion Control
Source: ACS Photonics. 2023 Aug 27;10(9):3223–32. doi: 10.1021/acsphotonics.3c00630 (PMC10515694; doi:10.1021/acsphotonics.3c00630)
Supplement: Supplementary file 1 — ph3c00630_si_001.pdf [file ph3c00630_si_001.pdf]

Supporting Information. Optically driven Janus microengine with full orbital motion control

David Bronte Ciriza,*,† Agnese Callegari,*,‡ Maria Grazia Donato,† Berk Çiçek,¶ Alessandro Magazzù,† Iryna Kasianiuk,¶,§ Denis Kasyanyuk,¶,§ Falko Schmidt,‖ Antonino Foti,† Pietro G. Gucciardi,† Giovanni Volpe,‡ Maurizio Lanza,† Luca Biancofiore,*,¶,§ and Onofrio M. Maragò†

†CNR-IPCF, Istituto per i Processi Chimico-Fisici, I-98158, Messina, Italy

‡Department of Physics, University of Gothenburg, SE-41296 Gothenburg, Sweden

¶Department of Mechanical Engineering, Bilkent University, TR-06800, Ankara, Turkey

§UNAM - National Nanotechnology Research Center and Institute of Materials Science & Nanotechnology, Bilkent University, 06800 Ankara, Turkey

‖Nanophotonic Systems Laboratory, Department of Mechanical and Process Engineering, ETH Zurich, CH-8092, Zurich, Switzerland

E-mail: brontecir@ipcf.cnr.it; agnese.callegari@physics.gu.se; luca@bilkent.edu.tr

Number of pages: 7

Number of videos: 3

Number of figures: 7

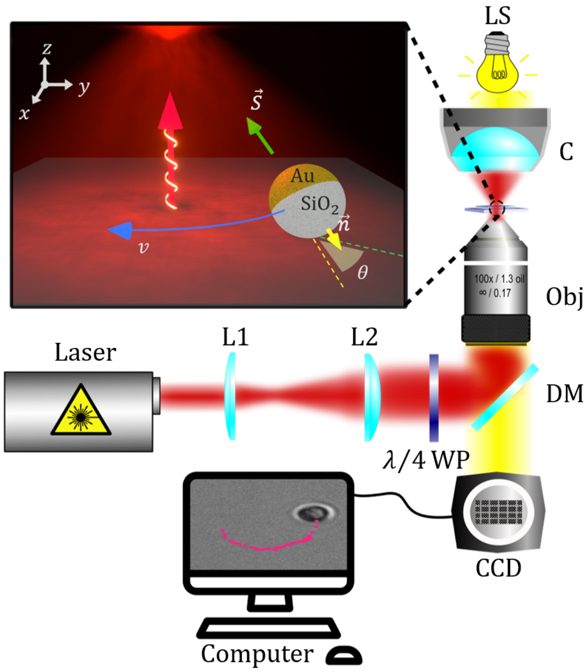

Fig. S1: **Schematic of the experimental set up.** The schematic includes the laser source, the two lenses (L1 and L2) that expand the beam, the $\lambda/4$ waveplate, the dicroic mirror (DM) that reflects the beam into the objective (Obj), the condenser (C), the illumination source (LS), and the computer that analyzes the images.

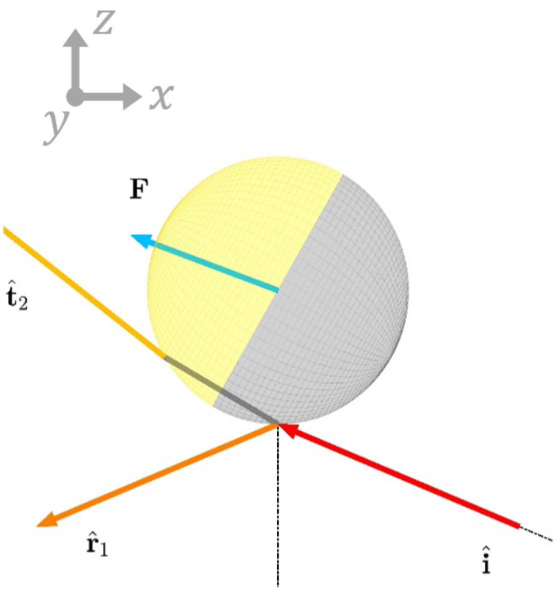

Fig. S2: **Schematic of a ray impinging on a Janus particle.** The ray $\hat{\mathbf{i}}$ reaches the particle and divides into a scattered ray $\hat{\mathbf{r}}_1$ and a transmitted ray $\hat{\mathbf{t}}_2$. The change in linear momentum results in an applied force on the particle $\mathbf{F}$

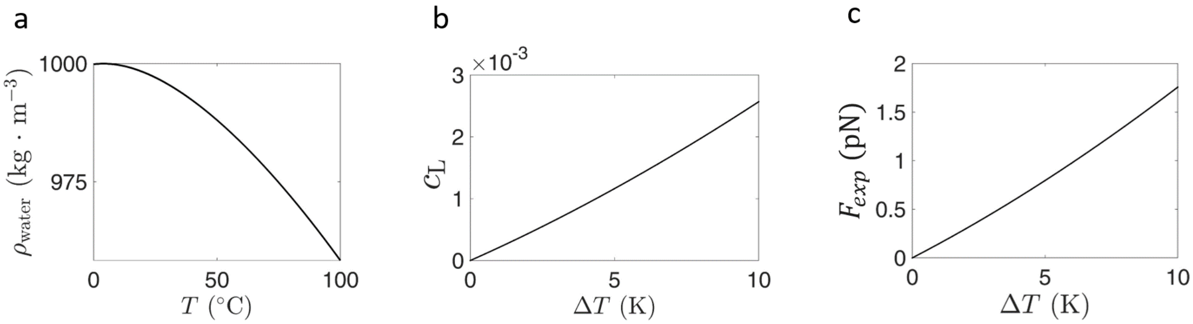

Fig. S3: **Density of water (ρ_{water}), linear expansion coefficient of water (c_L) and volume expansion force (F_{exp}) as a function of temperature.**

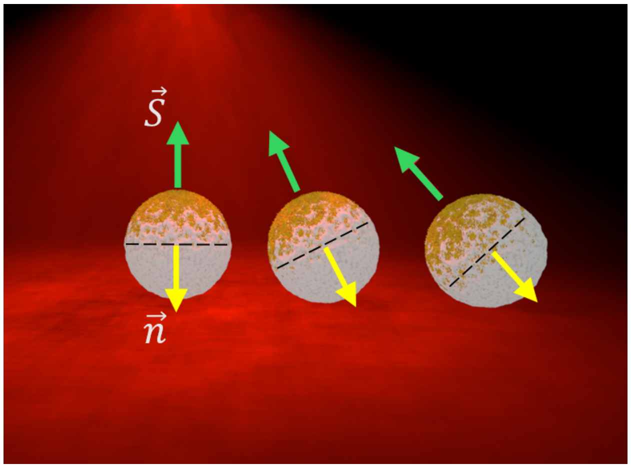

Fig. S4: **Orientation of the Janus particle for different radial positions under linearly polarized light.** The local Poynting vector of the focused beam (S, in green) is perpendicular to plane that contains the border between gold and silica and goes in the opposite direction to the the orientation vector ($\vec{n}$, in yellow).

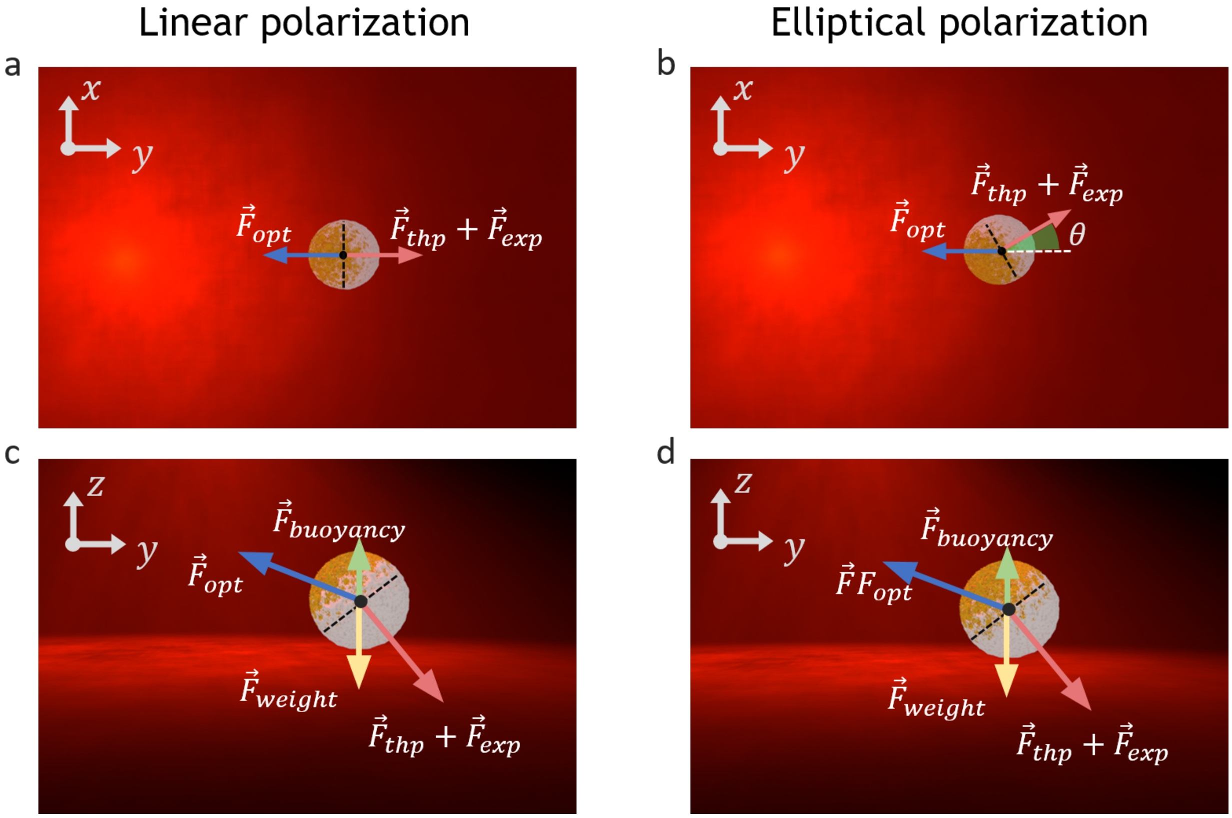

Fig. S5: **Forces acting on the Janus particle under different laser polarizations.** Forces acting on the Janus particle under linearly polarized light (a,c) and elliptically polarized light (b,d). The optical force acting on the particle can be decomposed into two components: a gradient part that directs the particle towards the center, and a scattering part in the z direction, which pushes the particle away from the cover slip. In (b), θ is the angle between the direction of the optical force (towards the center of the beam) and the direction of the thermal force (from gold to silica) that gives rise to the tangential force that drives the orbital motion.

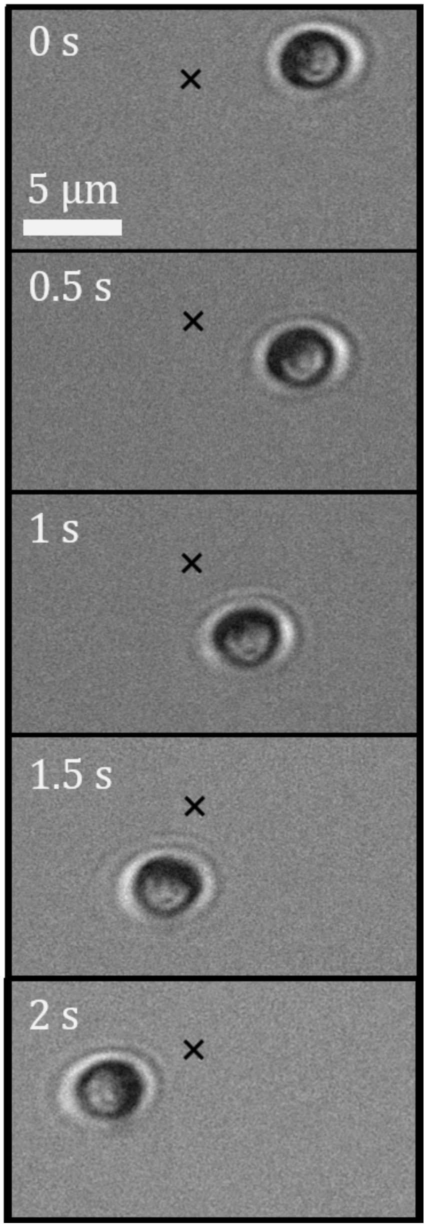

Fig. S6: **Orientation of the Janus particle under circularly polarized light.** Janus particle rotating under light circularly polarized clockwise. The particle is shown at $t = 0, 0.5, 1, 1.5$ and 2 s. The black cross indicates the centre of the beam. The gold-coated side of the Janus particle (the darkest region in transmission microscopy) faces always radially inwards to the center of the beam while the silica particle (lightest region) faces outwards.

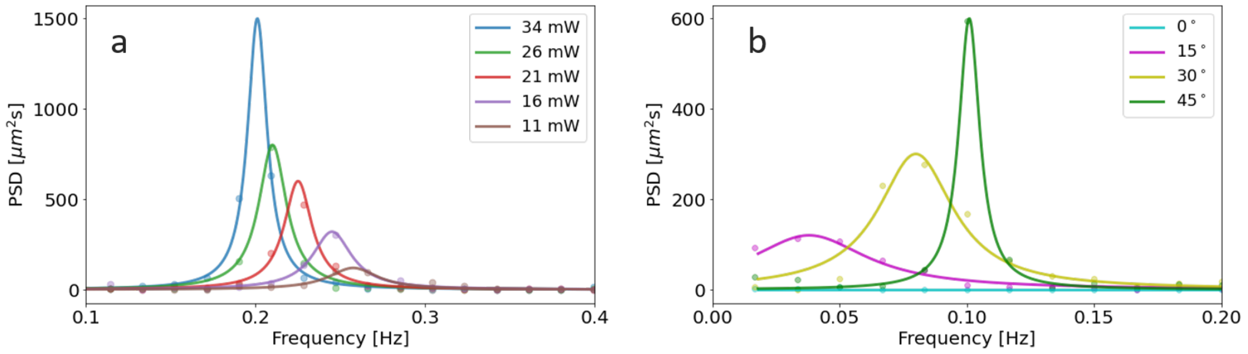

Fig. S7: **Power spectral density of the trajectories of the microengine.** (a) Light circularly polarized and with different powers. (b) Light with constant power (34 mW) and different degrees of ellipticity of the incoming light.
